# May-Hegglin anomaly associated nephropathy: Case series

**DOI:** 10.1177/2050313X241302013

**Published:** 2024-11-25

**Authors:** Matthew D Nguyen, Gayathri Dileep, Marrey Quizon, Vu Nguyen, Arif Demerci, Ramy Hanna

**Affiliations:** Division of Nephrology, Hypertension and Transplant Nephrology, University of California, Irvine, CA, USA

**Keywords:** Myh9, May-Hegglin anomaly, chronic kidney disease, nephropathy, thrombocytopenia, Myh9-RD

## Abstract

May-Hegglin anomaly (MHA) is a rare autosomal dominant disease associated with a mutation in the MYH-9 gene. It is characterized by macrothrombocytopenia and neutrophils with abnormal cytoplasmic inclusions. Clinical features of this disease include hearing loss, early cataracts, and renal failure. We present two interesting cases of renal injury attributed to MHA. The first is a 52-year-old Hispanic female with MHA-associated nephropathy and thrombocytopenia complicated by Stage 3 chronic kidney disease (CKD) and hypothyroidism. She was found to have a pathogenic MYH-9 heterozygous mutation with associated clinical characteristics. Due to her thrombocytopenia from MHA, patient is not a candidate for kidney biopsy. She has been treated with SGLT-2 inhibitors, Angiotensin Receptor Blockers (ARBs) for managing her Stage 3b CKD and Synthroid^®^ for hypothyroidism. Despite these treatments, she continues to have low platelet counts, proteinuria, and progressive CKD. Our second case highlights a 39-year-old white female with MHA associated with focal segmental glomerulosclerosis diagnosed at the age of 15 on renal biopsy. She also has thrombocytopenia and mixed connective tissue disease with rheumatoid arthritis and systemic lupus erythematosus clinical characteristics. She is currently on a regimen of methotrexate, Xeljanz, and IVIG for her rheumatological diseases. Her kidney function has remained stable on Angiotensin Converting Enzyme Inhibitors (ACEi) with hydrochlorothiazide and as needed loop diuretics for edema. These cases illustrate the challenges of diagnosing and managing renal complications associated with MHA due to the MYH-9 gene mutation. Chronic thrombocytopenia in both patients restricts the use of invasive diagnostic procedures, such as biopsies, which are critical for confirming the relationship between nephropathy and MHA, and for guiding further treatment. As such, these cases stress the importance of genetic testing as a key tool in diagnosis and emphasize the difficulties in managing patients with suspected MHA-associated nephropathy and thrombocytopenia.

## Introduction

The rare autosomal dominant disease associated with the MYH-9 gene mutation is May-Hegglin anomaly (MHA). Currently, the incidence and prevalence worldwide are unknown; however, fewer than a hundred cases have been reported in the literature.^1,2^ It is characterized by neutrophils with abnormal cytoplasmic inclusions, large platelets, and variable thrombocytopenia.^3,4^ The proposed etiology involves a mutation on the MYH-9 gene located on chromosome 22q12-13, which encodes at a non-muscle myosin heavy chain class IIA, leading to its abnormal production. This abnormal production results in macrothrombocytopenia due to impaired megakaryocytic maturation and fragmentation. Despite these deficiencies, neutrophil and platelet dysfunction are considered to be normal.^1,5^

The MYH-9 gene is also implicated in MYH9-related diseases (MYH9-RD) which include the following diseases: MHA, Fechtner Syndrome, Sebastian Syndrome, and Epstein Syndrome.^6–8^ These disorders all share the characteristics of macrothrombocytopenia and characteristic leukocyte inclusions and are classified as autosomal dominant platelet disorders.^8^ Common clinical symptoms include sensorineural hearing loss, presenile (early) cataracts, and renal failure due to nephropathy.^6,7,9^

In this report, we present two cases of MYH9-RD-associated nephropathy. Our first case is a 52-year-old female with diagnosed MHA-associated nephropathy and thrombocytopenia, complicated by Stage 3 chronic kidney disease (CKD) and hypothyroidism. She was found to have a pathogenic MYH-9 heterozygous mutation on genetic testing, and now exhibits progressive proteinuria and renal function decline. Our second case depicts a 39-year-old white female with MHA associated with focal segmental glomerularsclerosis (FSGS), whose renal function has been stabilized with pharmacological therapy.

## Case presentation

### Case 1

**Figure 1.**
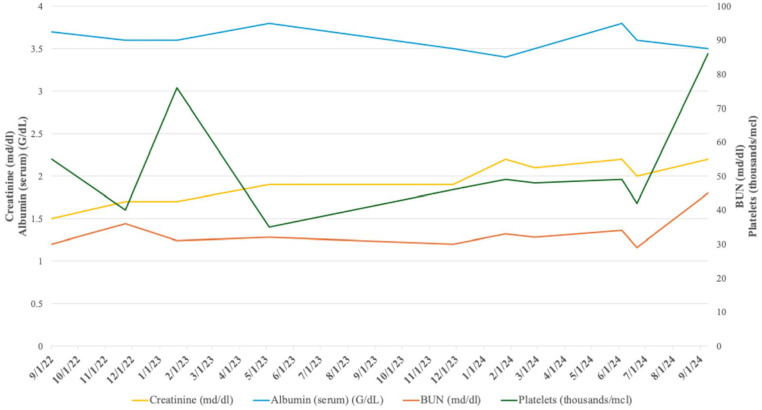
Case #1: Renal function.

Our first case is a 52-year-old Hispanic female diagnosed with MHA, hypothyroidism, CKD at an outside hospital and was referred to us for evaluation of Stage 3b CKD. During the initial visit, she reported not having any hearing loss or vision problems. Regarding her MHA history, she was diagnosed with MHA while pregnant with her first child at 27 years old, though she had exhibited symptoms earlier. For instance, at 7 years old, she experienced severe bleeding following an appendectomy, and at 13, she had a prolonged recovery after a tonsillectomy. She also reported that both her son and daughter also have MHA, with her daughter recently requiring a platelet transfusion. Both of her children were born vaginally without major complications, although there was a near-surgical event during her daughter’s birth (Figure 1).

When the patient first presented to our institution, she was on Losartan for hypertension and Synthroid for her hypothyroidism. She was also receiving antidiuretic hormone treatment. Her initial serum creatinine (sCr) was 1.7 mg/dl (0.5–1.2 reference range), with a glomerular filtration rate (GFR) between 30 and 44 ml/min/1.73 m² due to CKD Stage G3b/A1 and a urine albumin/creatinine ratio less than 30 mg/g. Her liver functions were normal. Following this visit, she was started on a sodium-dependent glucose cotransporter 2 (SGLT-2) inhibitors (Farxiga^®^) and advised to follow a renal diet due to her elevated body mass index (BMI) of 40.58 kg/m². Her kidney size was normal on ultrasound. At this time, we were hesitant to conduct a biopsy due to her MHA and concurrent low platelets.

At her second visit, her BMI had decreased to 38.62 kg/m². Her sCr remained at 1.7 mg/dl (0.5–1.2 reference range), B-type natriuretic peptide (BNP) was 103 pg/ml (0–100 pg/ml reference range), GFR was between 32 and 39 ml/min/1.73 m², serum blood urea nitrogen (sBUN) was 31 mg/dl (7–25 reference range), serum albumin (sALB) was 3.6 g/dl (3.7–5.3 reference range), and her 24 h urine total protein was 6.174 g/day (50–80 mg/day reference range). She still had thrombocytopenia with a platelet count of 76 k/μl (150–400 k/μl reference range), and a peripheral smear showed large platelets. Her anti-phospholipase A2 receptor (anti-PLA2R), anti-thrombospondin type 1 domain 7 (anti-THSD7), anti-nuclear antibody (ANA), anti-neutrophil cytoplasmic Ab (ANCA), anti-double stranded DNA (anti-DS-DNA), hepatitis panel, and human immunodeficiency virus (HIV) panel were negative. Her C3, C4, and CH50 were in normal range. Genetic testing was planned, and the biopsy was again withheld due to low platelet count. She remained on Losartan, Farxiga, and Synthroid.

At her third visit, her BMI had further decreased to 37.56 kg/m². Genetic testing results confirmed nephropathy due to her MHA. She continued to have low platelets and was still not a candidate for kidney biopsy. Her proteinuria was progressively worsening. At this time, the patient was advised to have her children undergo genetic testing for MHA. She stated that her children were experiencing symptoms of MHA, but had not yet developed proteinuria. She also reported no noticeable weight loss while on Farxiga. Her labs at this time showed sCr at 1.9 mg/dl (0.5–1.2 reference range), BNP at 116 pg/ml (0–100 pg/ml reference range), GFR between 28 and 34 ml/min/1.73 m², sBUN 32 mg/dl (7–25 reference range), and sALB at 3.8 g/dl (3.7–5.3 reference range). Her thrombocytopenia had worsened, with a platelet count of 35 k/μl (150–400 k/μl reference range), and her smear still showed large platelets. A renal ultrasound revealed no significant renal artery stenosis by Doppler parameters. Patient remained on Losartan, Farxiga (SGLT-2 inhibitor), and Synthroid, and was referred for genetic counseling.

### Case 2

**Figure 2.**
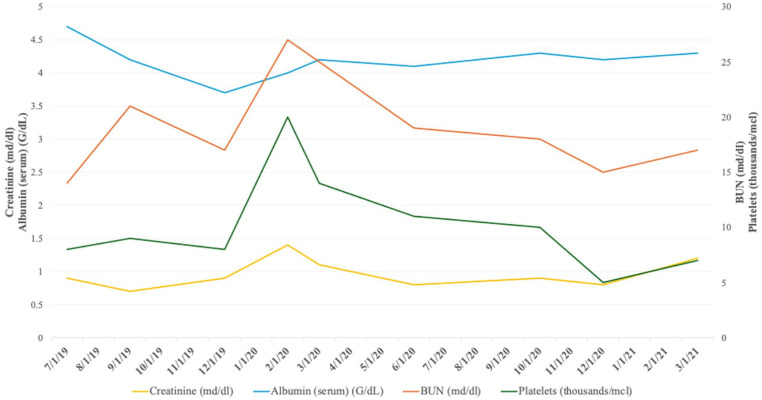
Case #2: Renal function.

Our second case is a 39-year-old white female with a history of MHA requiring multiple platelet transfusions and IVIG, undifferentiated connective tissue disorders, multiple rheumatologic disorders, autoimmune hepatitis, and Hashimoto’s thyroiditis who was referred to our institution for worsening proteinuria and renal function secondary to focal segmental glomerular sclerosis (FSGS). She was born with thrombocytopenia, which at the time was diagnosed as idiopathic thrombocytopenia purpura. However, her thrombocytopenia persisted throughout childhood. At age 15, she developed worsening proteinuria, and a renal biopsy revealed FSGS. In her 20s, she experienced hearing loss and was diagnosed with mixed connective tissue disease with rheumatoid arthritis and systemic lupus erythematous characteristics. Serological markers were notable for positive anti-smooth muscle antibodies, but all other titers were negative (including ANA, subsets, Rheumatoid Factor (RF), and complement levels). In her mid-30s, she developed the aforementioned collection of autoimmune disorders, and she was found to have a mutation in the MH&9 gene (Figure 2).

Upon her arrival at our institution, she was on Xeljanz, IVIG, enalapril, and hydrochlorothiazide for her hypertension, metformin for her diabetes, and methotrexate. At that time, she had ongoing 1 g of proteinuria, and her serum creatinine (sCR) has risen from 0.8 to 1 mg/dl (0.5–1.2 reference range) to 1.4 mg/dl, GFR between 30 and 44 ml/min/1.73 m², albuminuria creatinine ratio greater than 30 mg/g, and urine protein creatine ratio (UPCR) at 1 g. Her liver functions were normal. After her first visit, a repeat chemistry panel was ordered due to suspicion of pre-renal or medication-related effects. The dose of Xeljanz was reduced where possible due to its association with elevated sCr, and IVIG without sucrose was recommended. Enalapril was continued as tolerated by the patient. A repeat renal biopsy was not performed due to her low platelet count, which was 20 k/μl.

Over the next subsequent visits, her sCR decreased to 0.7 mg/dl (0.5–1.2 reference range) and remained stable. Her UPCR remained consistently at 1 g, well within the expected range for secondary FSGS. Systolic blood pressure continued to be above 120 mmHg. With the continuing reoccurrence of edema, hydrochlorothiazide was continued along with a loop diuretic. Potassium supplementation was also initiated prophylactically, given concerns for hypokalemia. Her renal function had remained stable with pharmacological therapy since the most recent visit. At this time, we suspect that the FSGS may be secondary to her MHA or other genetic factors; however, we are unable to confirm this suspicion due to the inability to perform a repeat biopsy because of her chronically low platelet levels.

## Discussion

The heavy chain of non-muscle myosin IIA, coded by the MYH-9 gene, generates intracellular chemo-mechanical forces that participate in various processes requiring displacement of the actin cytoskeleton.^10^ When a mutation occurs in the MYH-9 gene, it causes MYH9-RD, one of which is MHA.^7^ Currently, there is a paucity of literature on MHA, especially regarding its effects on renal function. To address this gap, we present these two interesting cases to highlight the challenges in diagnosing and managing this disease.

The association, incidence, and prevalence of nephropathy in MYH9-related disorders are understudied. In a case series by Oh et al., nephropathy was detected in 30% of MYH9-related disorder cases, with characteristic features definitive of early-onset proteinuria and rapidly progressing renal disease, similar to our own patient cases.^11^ Furthermore, another study by Pecci et al. also reported that 61 of 247 patients (roughly 25%) developed proteinuria and nephropathy.^12^ All of these studies, including our case series, incite further evaluation for the epidemiological bassline of MHA and associated nephropathy.

In both cases, diagnosing nephropathy was challenging due to thrombocytopenia, which limits the use of invasive diagnostic modalities such as renal biopsy. Steroid medications for thrombocytopenia were withheld as contraindicated in our FSGS patient. At this time, treatment of CKD progression secondary to MYH-9-related nephropathy is limited in study. Thus, current treatment modalities primarily focus on reducing proteinuria and preserving renal function by inhibiting the renin-angiotensin system, as we emulated in our two cases.^11,13,14^

In both cases presented, we documented the progression from diagnosis of MHA to CKD and the management of progressive CKD. MHA is a highly rare condition, and diagnosing it, especially when nephropathy is involved, remains a significant challenge. We suggest that clinicians conduct genetic testing before diagnosing to rule out autoimmune diseases. However, further investigations are needed to explore the role of the MYH9 gene and its relationship to renal function.
