# Corrigendum to “May-Hegglin anomaly associated nephropathy: Case series”

**DOI:** 10.1177/2050313X241309485

**Published:** 2024-12-17

Nguyen MD, Dileep G, Quizon M, Nguyen V, Demerci AN, Hanna R. May-Hegglin anomaly associated nephropathy: Case series. *SAGE Open Med Case Rep.* 2024;12. doi:10.1177/2050313X241302013

In the above-mentioned article, the name of the author has been changed from “Arif Demirci” to “Arif Nihat Demirci”.

The article has been updated to reflect the correction.

